# Alignment and Prediction of *cis*-Regulatory Modules Based on a Probabilistic Model of Evolution

**DOI:** 10.1371/journal.pcbi.1000299

**Published:** 2009-03-13

**Authors:** Xin He, Xu Ling, Saurabh Sinha

**Affiliations:** Department of Computer Science, University of Illinois Urbana-Champaign, Urbana, Illinois, United States of America; University of British Columbia, Canada

## Abstract

Cross-species comparison has emerged as a powerful paradigm for predicting *cis*-regulatory modules (CRMs) and understanding their evolution. The comparison requires reliable sequence alignment, which remains a challenging task for less conserved noncoding sequences. Furthermore, the existing models of DNA sequence evolution generally do not explicitly treat the special properties of CRM sequences. To address these limitations, we propose a model of CRM evolution that captures different modes of evolution of functional transcription factor binding sites (TFBSs) and the background sequences. A particularly novel aspect of our work is a probabilistic model of gains and losses of TFBSs, a process being recognized as an important part of regulatory sequence evolution. We present a computational framework that uses this model to solve the problems of CRM alignment and prediction. Our alignment method is similar to existing methods of statistical alignment but uses the conserved binding sites to improve alignment. Our CRM prediction method deals with the inherent uncertainties of binding site annotations and sequence alignment in a probabilistic framework. In simulated as well as real data, we demonstrate that our program is able to improve both alignment and prediction of CRM sequences over several state-of-the-art methods. Finally, we used alignments produced by our program to study binding site conservation in genome-wide binding data of key transcription factors in the *Drosophila* blastoderm, with two intriguing results: (i) the factor-bound sequences are under strong evolutionary constraints even if their neighboring genes are not expressed in the blastoderm and (ii) binding sites in distal bound sequences (relative to transcription start sites) tend to be more conserved than those in proximal regions. Our approach is implemented as software, EMMA (Evolutionary Model-based *cis*-regulatory Module Analysis), ready to be applied in a broad biological context.

## Author Summary

Comparison of noncoding DNA sequences across species has the potential to significantly improve our understanding of gene regulation and our ability to annotate regulatory regions of the genome. This potential is evident from recent publications analyzing 12 *Drosophila* genomes for regulatory annotation. However, because noncoding sequences are much less structured than coding sequences, their interspecies comparison presents technical challenges, such as ambiguity about how to align them and how to predict transcription factor binding sites, which are the fundamental units that make up regulatory sequences. This article describes how to build an integrated probabilistic framework that performs alignment and binding site prediction simultaneously, in the process improving the accuracy of both tasks. It defines a stochastic model for the evolution of entire “*cis*-regulatory modules,” with its highlight being a novel theoretical treatment of the commonly observed loss and gain of binding sites during evolution. This new evolutionary model forms the backbone of newly developed software for the prediction of new *cis*-regulatory modules, alignment of known modules to elucidate general principles of *cis*-regulatory evolution, or both. The new software is demonstrated to provide benefits in performance of these two crucial genomics tasks.

## Introduction

The spatial-temporal expression pattern of a gene is controlled by its regulatory sequences, sometimes called a *cis*-regulatory module (CRM). A CRM contains a number of transcription factor binding sites (TFBSs), which read the expression level of the cognate transcription factors (TFs) and drive the appropriate expression pattern through the combinatorial interactions among TFs, their co-factors and the basal transcriptional machinery [1]. Cross-species comparison plays a central role in various problems involving *cis*-regulatory sequences, including computational prediction of CRM sequences [2]–[4], discovery of novel sequence motifs [5],[6] and exploration of the principles of regulatory sequence evolution [7]. For these different types of analysis, the standard procedure almost always starts with an alignment of these sequences, followed by an analysis of the conservation pattern of sequences as suited for the specific task.

The first major limitation of this two-step procedure arises from errors in alignment. It has been shown that alignment procedure may seriously affect the results of comparative genomic analysis such as reconstruction of phylogenetic trees and inference of positive selection [8]. Most alignment tools are not customized to regulatory sequences, and thus cannot take advantage of their specific structural and evolutionary properties. A second shortcoming of many current methods for regulatory sequence comparison is their heuristic nature. It is difficult to assess the significance of the results if appropriate statistical models have not been specified and used. While there are indeed a number of successful programs based on sound statistical models of DNA sequence evolution [9],[10], few of them incorporate the CRM structure. Finally, it is commonly assumed that a TFBS is conserved across all species being studied [5],[11]. However, there is strong evidence that functional noncoding sequences in general, and TFBSs in particular, are not always conserved in an alignable sequence even in relatively close species [12],[13]. This process of TFBS change has been recognized as an important source of evolution of phenotypes [14].

Several approaches have been proposed to address one or more of the problems discussed above. The programs Stubb [3], EvoPromoter [15] and PhylCRM [16] predict CRMs as significant clusters of TFBSs, which are detected by comparing orthologous sequences using an evolutionary model of binding sites. However, all methods require a fixed alignment as input and do not model the binding site gains and losses. The programs CONREAL [17], EEL [18] and SimAnn [19] align putative CRM sequences with the explicit goal of aligning the sites matching known TF profiles. None of these methods use rigorous statistical or evolutionary models, and they all assume the complete conservation of TFBSs. Moses et al. [13] deal with alignment uncertainty in their analysis of binding site turnover, but this is done as a post-hoc analysis rather than being integrated with the inference step. Our recent work, Morph [20], tries to solve all the above problems in a single framework with a pair-HMM model. However, the Morph model does not accurately capture the evolutionary dynamics of CRMs. Lineage specific TFBSs are treated not as gain or loss events in evolutionary time, but merely as HMM “emissions” from one sequence, and not the other. Another recent work, SAPF [21], aims to combine probabilistic model-based alignment with “phylogenetic footprinting”, which refers to the identification of evolutionarily constrained sequences based on their lower substitution rates. However, TFBSs are not explicitly represented in the SAPF model, and the program is not designed to predict targets of specific transcription factor(s). Our goal is not to detect constrained sequences per se, but the target sequences of specific transcription factors, whose binding motifs are known *a priori*.

Our philosophy of doing cross-species sequence analysis is: firstly, the method should be based on an explicit model of sequence evolution, as expressed in [10] – “the study of biological sequence data should not be divorced from the process that created it”; secondly, the problem should be solved in a single, integrative framework, instead of being split into multiple steps. Specifically, this means that to predict a CRM, one should take into account the uncertainty of alignment and TFBS annotation by summing over them from a combined statistical model. The above philosophy has been adopted previously in the area of statistical alignment [10],[22],[23], where stochastic models are used to describe the evolution of indels and the alignment task is often integrated with the ultimate goals, most notably, the reconstruction of phylogenetic tree [24]. Models that describe one or more aspect of regulatory sequence evolution have been proposed recently [25]–[31], but none of these methods offers a complete evolutionary model of CRM sequences that can be directly used for bioinformatic tasks such as CRM alignment and prediction. We propose an expressive and biologically realistic model of CRM evolution where (i) stochastic models of substitution and indels are used to characterize the evolution of background sequences (non-TFBS sequences inside a CRM); (ii) TFBSs evolve according to a population genetic model developed previously; (iii) functional switching between a non-TFBS and TFBS can occur in a manner dependent on the binding energy of the evolving site. We implement an efficient inference machinery and apply it to the tasks of CRM alignment and prediction.

We used alignments produced by our program to analyze the regulatory sequences involved in early development of *Drosphila melanogaster*. We took advantage of the recent genome-wide binding data on key TFs involved in blastoderm-stage gene regulation (obtained using ChIP-chip technology [32]), and tested two important hypothesis. First, we investigated previously published claims that there is a high level of non-functional binding in such genome-wide TF binding studies [33],[34]. The prime candidates of such non-functional binding sites are those that are not adjacent to genes expressed in blastoderm. If the claim is true, we expect that these sites will be less conserved than binding sites adjacent to appropriately expressed genes. We found statistical evidence to the contrary, opening up the possibility of functional binding at a larger scale than previously thought. Second, the positions of CRMs relative to the coding sequences, may have a large impact on their functions. For example, computationally predicted CRM sequences enriched with TFBSs are much more likely to drive expression of reporter genes if they are located close to transcription start site (TSS) [2]. Wray has suggested an interesting hypothesis that the CRMs near TSS are likely “control modules”, while those distal ones may be “booster modules” that are less essential [35]. We tested this hypothesis by comparing the conservation level of TFBSs in proximal bound regions and in distal ones. We find no support for this hypothesis, and in fact distal bound regions seem to have a greater conservation of binding sites than proximal regions, contrary to expectation.

## Results

### Evolutionary Model of *cis*-Regulatory Modules

In this section, we present the details of our model, which first captures the salient properties of a CRM's content and then lays out the evolutionary forces acting upon its different components. The model prescribes the joint likelihood of a set of orthologous CRMs that are related by a given phylogenetic tree.

We begin with a model of CRM composition and assume that the ancestral CRM is generated from this model. We use a generalized HMM of zero order, similar to the ones used in [3],[15],[36]. The binding specificities (motifs) of 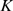 TFs are represented by 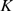 position weight matrices (PWMs), and the nucleotide frequencies of the background sequence are denoted by 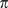. At each step, the background state or the 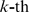 motif is sampled with probability 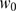 and 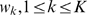, respectively. If the 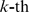 motif is chosen, the actual site is sampled from the 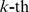 PWM; otherwise, a single nucleotide is sampled from 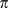. The HMM transition probability, 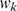, can be interpreted as the average number of binding sites of this motif per nucleotide at equilibrium, or simply binding site density.

Our evolutionary model of the background sequences is adapted from the models developed earlier for “statistical alignment” [9],[22],[37]. Substitutions are described by the standard HKY model [38], with equilibrium distribution 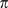 and transition-transversion bias 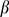. Insertions and deletions follow Poisson processes with rates 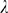 and 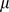 respectively. The length of an indel follows the geometric distribution with the probability of extension 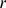. Following this model, the joint probability of the sequences 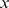 and 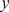 in the example below, under a two-species phylogenetic tree with branch lengths 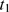 and 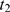, is approximately (# stands for any nucleotide):
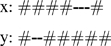


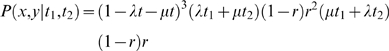
(1)where 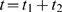. The terms 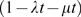, 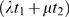 and 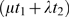 are the probabilities of not seeing an indel event in 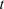, of seeing a gap in the second sequence and of seeing a gap in the first sequence, respectively. The derivation of these probabilities can be found in Text S1.

We use the population genetics-based Halpern-Bruno (HB) model for TFBS evolution [39],[40]. This model captures the fact that the evolutionary constraints at different positions of a TFBS may be different: less degenerate positions in the PWM generally have lower substitution rates. Let 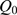 be the substitution rate matrix of the background sequences, and 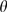 be the PWM of the motif being evolved, the rate of substitution of a nucleotide 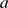 to 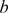 at position 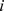 is:
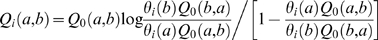
(2)The transition probability of 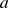 to 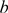 in time 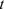 is thus the 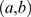 entry of the matrix 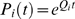. Since HB model is time reversible, the joint probability: 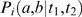 is simply 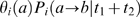.

Gain and loss of TFBSs are commonly observed across a large evolutionary spectrum: e.g. fungi [41], insects [13] and vertebrates [12]. There are two different scenarios in which these events may occur. In the first scenario, the expression pattern of the target gene is under adaptive change, which “demands” a change in the composition of the controlling CRM, causing binding site gains and losses. In the second scenario, the expression of the gene is under stabilizing selection, but the selection on individual TFBSs may be weak, and as a result, a TFBS may be lost during evolution due to random drift. New TFBSs may also be created in the background sequences simply by mutations and random drift, due to the fact that TFBSs are often short and degenerate. The two processes may be linked to each other: the loss of one TFBS could make gain of a TFBS in the background more beneficial so as to compensate for the loss; likewise the gain of a new site could make existing sites redundant, thus relax the constraints and speed up the loss process.

The main difference between the two scenarios is: in the former, the changes of TFBSs are driven by external selection forces while in the latter, the changes are mainly dominated by the stochastic forces of mutation and random drift, with selection being weak. In our model, we adopt the second scenario as it is a more “parsimonious” explanation of TFBS gain and loss, and is more consistent with our current knowledge about the *Drosophila* early developmental CRMs [7],[42], which are among the most well-characterized available today.

Our specific model formulates the ideas discussed above. We follow the usual definition of binding energy of a TFBS, for example [28]. We assume that there is a threshold for the binding energy of a site, 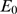, above which a site is not functional. We use 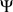 and 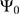 to denote the evolutionary models of a TFBS and non-TFBS respectively. Our basic idea for modeling gain and loss is: switching of a site between TFBS and non-TFBS states is a switch between the models that govern the evolution of this site. Under the model 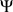, mutations that change the energy of a site above 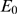 may occasionally be fixed due to random drift. After that point, natural selection will not be able to perceive this site (switch to 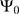). Likewise, under the model 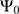, a background site could occasionally reach 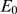 by mutation and random drift. This site will then be visible to its cognate TF and will be subjected to natural selection (switch to 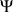). We note that indel events may happen inside TFBSs, albeit with a much lower rate than in background sequences, and we denote by 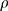 the relative rate of intra-TFBS indels. Interaction between gain and loss events as explained above is not explicitly modeled, to avoid creating dependencies that make the computational task much more difficult.

**Figure 1:**
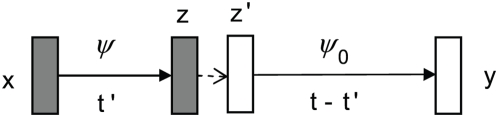
A model of TFBS loss. Shaded and white box represent functional TFBS and non-functional site respectively. The dashed arrow between 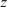 and 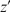 indicates the instantaneous substitution or indel event that disrupts the site.

We illustrate our model of TFBS loss in Figure 1: starting with a functional site 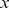, a substitution or indel event disrupts this site at time 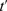; the background model then governs the evolution of this site, which eventually becomes sequence 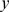. Let 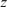 and 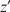 be the sequences preceding and following the loss event respectively, then:
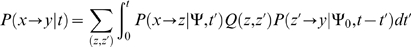
(3)where 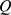 is the instantaneous rate of substitution (given by Eq. 2) or indel (given by the product of 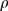 and the background indel rate) under the model 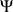, the evolutionary model of TFBSs; and 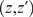 must satisfy the energy constraint: 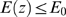 and 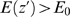, and the neighborhood constraint: they differ by a single mutation event. The probability of TFBS gain can be calculate in a similar way. Joint probability under a two-species phylogenetic tree can be found in Text S1 and Figure S1. For computational efficiency, we make the parsimony assumption: suppose 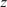 is an intermediate site between 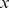 and 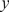, then the symbol at any position of 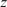 is either the symbol of 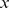 or of 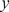 at that position. We also note that, even though we rely on a threshold for determining when binding site gain or loss happens, this parameter is not directly used for classifying a site as functional or not. Instead, the annotation of a site depends upon an examination of the site and its orthologous sequences, and their probability under different histories: background, conserved or lineage-specific.

### Statistical Inference

We solve the following computational problems: given two orthologous sequences (that are roughly alignable so that they could be identified in the first place) and a set of TF motifs, (1) align the two sequences and annotate the TFBSs; (2) predict if the sequence is a CRM targeted by the given motifs. We use dynamic programming to simultaneously find the optimal alignment and TFBS annotation. For the second task, we use a likelihood-ratio test of two models: the CRM evolutionary model and the background evolutionary model where no motif is used. Computation under each model is also done by dynamic programming, summing over all possible alignment paths and annotations of TFBSs. The details of the algorithms can be found in Materials and Methods. We allow all parameters to be learned automatically from the data while allowing certain parameters to be specified by users (Text S1). Our computational framework is implemented as a program called EMMA (Evolutionary Model-based *cis*-regulatory Module Analysis).

### Comparison of Alignment Methods Using Simulated Data

Simulation of sequence evolution is a useful strategy for assessing computational methods, as the true evolutionary history is often unknown for real data. In addition, simulation is an important way to understand how various factors, such as divergence time or the presence of TFBSs, affect the performance of a computational procedure, since such questions are generally difficult to answer analytically. We developed a simulation program that can generate orthologous CRM sequences according to our evolutionary model. The binding site densities, branch lengths, indel rates, etc., are all user-specified parameters. Our simulator captures a richer biology of regulatory evolution than many other sequence simulators [43],[44] and can be used as a general tool for the study of *cis*-regulatory evolution.

We first used simulation to study alignment methods, similar to what was done previously by Pollard et al. [44] and Huang et al. [45]. We implemented several versions of EMMA so that we could study the effect of each of its features. We denote by EMMA0 the version that uses only the background model, but not the motifs (thus EMMA0 is equivalent to the traditional Needleman-Wunsch alignment with affine gap penalty); EMMA1 is the version that considers only conserved TFBSs; and EMMA2 models both conserved and lineage-specific TFBSs. In addition, we tested a widely-used general-purpose alignment tool, Lagan [46], and our recently developed program for aligning regulatory sequences, Morph [20]. Morph uses a pair-HMM to model the alignment of two sequence, where the HMM contains several motif states to encode the presence of TFBSs in one or both sequences. We note that Morph does not model binding site turnover, so a non-conserved site will be aligned with gaps, instead of its true orthologous sequence. All programs were run under the default settings. The alignment performance was measured using specificity and sensitivity, as in [20]. In addition, we defined a measure called “TFBS conservation sensitivity” as the percentage of all positions in conserved TFBSs that are correctly aligned. For CRMs, this is clearly a more relevant measure of alignment quality [44].

**Figure 2:**
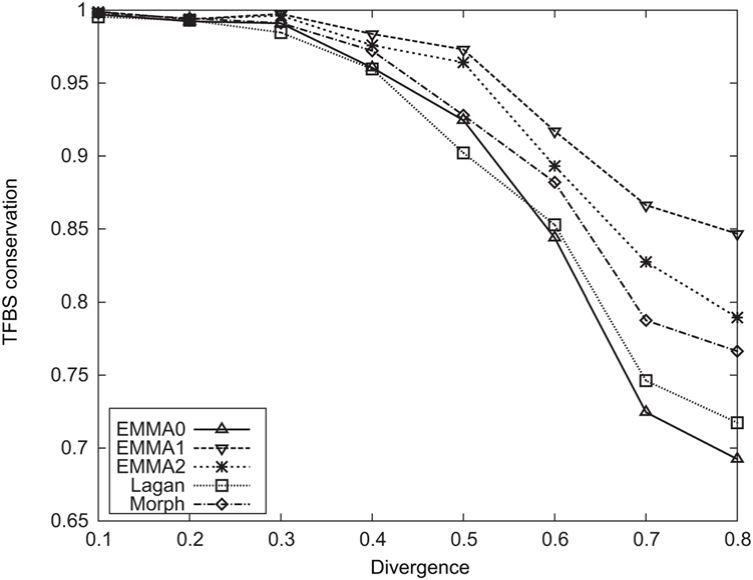
TFBS conservation sensitivity from various alignment programs using simulated data.

The specificities and sensitivities of all programs are similar (see Tables S2 and Table S3 in Text S1) because different programs differ mostly in treating TFBSs, which occupy a small fraction of the total sequence length. The results with the TFBS conservation measure are shown in Figure 2. EMMA0 and Lagan have similar performance with all three measures. This suggests that the values of alignment parameters have relatively small effect on the alignment quality. At moderate to high divergences, both EMMA1 and EMMA2 significantly outperform EMMA0 and Lagan in terms of TFBS conservation sensitivity (e.g. EMMA1 is better than Lagan by 12% and 13% respectively at divergence 0.7 and 0.8) and are slightly better with the other two measures, suggesting that modeling conserved TFBSs is beneficial to alignment of divergent sequences. Modeling lineage-specific TFBSs does not seem to help alignment, as EMMA1 is slightly better than EMMA2 at high divergence levels. This somewhat counter-intuitive observation may be explained by the fact that in pairwise comparison, lineage-specific TFBSs will not help alignment by serving as “anchors”; on the other hand, a truly-conserved TFBS may occasionally be treated as two lineage-specific sites in EMMA2. Morph is superior to Lagan and EMMA0 in terms of aligning conserved TFBSs, but not as good as EMMA1 and EMMA2. In addition, the higher TFBS conservation of Morph is achieved at the cost of significantly lower overall alignment sensitivity (more than 6% lower than all other programs at divergence greater than 0.5, see Tables S2 and S3 in Text S1).

Pollard and colleagues also studied the problem of how alignment affects binding site detection, through simulation [44],[47]. Their recent program, CisEvolver [44], is similar to our simulator in that both treat sequences as a mixture of background and TFBSs, and both use the Halpern-Bruno model for binding site evolution. The main differences include: CisEvolver uses empirical indel frequencies to parameterize the evolution of indels while we use a simpler geometric distribution; and CisEvolver does not incorporate the possible gain and loss of functional sites. Nevertheless, to test the robustness of our conclusions, we generated the test data using CisEvolver and repeated the comparisons described above. The results are in broad agreement with our previous results (see Figure S2, and Tables S4 and S5 in Text S1), suggesting that EMMA is robust to the treatment of indels and that our gain and loss model will perform well even if there are actually no or few gain or loss events (i.e., it will not introduce such events artificially). The PSPE program [45] is also capable of simulating the evolution of CRMs. It goes beyond modeling the gain and loss of individual binding sites, and captures features not available in either our simulator or CisEvolver; for example, it allows “global” fitness constraints such as “the total number of sites in a CRM must fall in some range”. However, its semantics of sequence alignment is different from the conventional notion of alignment (i.e., nucleotide-level orthology), making its benchmarks unsuitable for studying our program's performance. Furthermore, these alignment benchmarks were obtained under the assumption that each TF had one and only one site in a CRM, an assumption that is overly stringent given that many factors are known for homotypic clustering [48]. Also, the key idea of the PSPE model is “replacement turnover”, where one site loss exactly matches a gain in another site and vice versa, but it is not clear if this is a general evolutionary process. One recent study did not find that such change is important in explaining the observed patterns of binding site turnover [13]. Based on these considerations, we chose not to test EMMA on the benchmark data of PSPE.

### Comparison of CRM Prediction Methods Using Simulated Data

We investigated the problem of predicting CRM sequences given a set of motifs, again using simulated sequences. We implemented another program called EMMA-ANN, which scores a sequence by its conserved TFBSs using a fixed, non-motif alignment (we used EMMA0 alignment). The only difference between EMMA-ANN and EMMA1 is that a fixed alignment is used in the former, so the comparison between the two should suggest how important it is to treat alignment uncertainty. In addition to the EMMA family programs and Morph, we tested the program Stubb [3], which scores a sequence by its binding site cluster while favoring the conserved sites. Similar to EMMA-ANN, Stubb is also based on a fixed alignment. Here, a site that matches the PWM, but does not fall inside an aligned region, is allowed to contribute to CRM scoring; in contrast, only conserved sites are allowed to make contributions in EMMA-ANN. Thus in terms of identifying binding sites, Stubb is likely to be more sensitive but less accurate, a feature that has implications on CRM prediction. We generated a positive set of sequences simulated under the CRM evolutionary model, and a negative set under the background model. A program is tested by its ability to discriminate positive and negative sequences: all the sequences will be scored by the program; as the score threshold varies, the specificity and sensitivity will be computed. The overall performance of the program is measured by the area under curve (AUC) of the ROC curve, i.e., the plot of sensitivity vs (1 - specificity).

**Figure 3:**
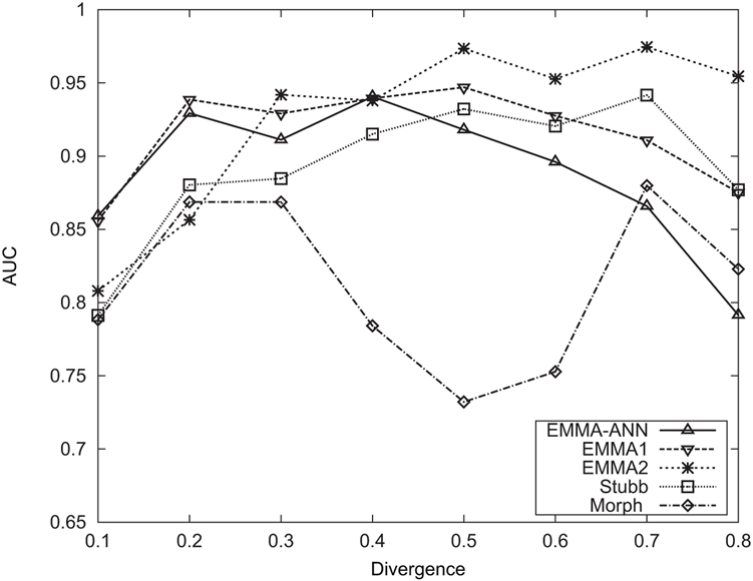
AUCs of different programs for CRM classification using simulated data.

The results are shown in Figure 3. The first observation is that high accuracy of prediction can be achieved even at relatively large divergence. Our explanation is that at higher divergence, conserved TFBSs will be more significant, i.e., less likely to be explained by the chance conservation of neutral sequences. The implication is that CRM prediction is sensitive to the correct alignment of conserved TFBSs, but not to the overall alignment quality. Comparisons between EMMA1 and EMMA-ANN suggest that simultaneous alignment and CRM scoring consistently improve prediction at almost all levels of divergence examined. The effect of modeling lineage-specific TFBSs, as seen from the comparison between EMMA1 and EMMA2, is somewhat mixed: at lower divergence (<0.3), it reduces the performance by 5–8%; at higher divergence, it improves by 3–8%. The intuition is: TFBS gain and loss will become common and thus important for the algorithms only at relatively high level of divergence. EMMA2 is consistently better than both Stubb and Morph which are based on phenomenological models (e.g. at divergence 0.8, AUC of EMMA2 is 0.95, while AUCs of Stubb and Morph are 0.87 and 0.82 respectively), suggesting the importance of correct models. Morph unexpectedly shows poorer performance than all other methods including Stubb. The underlying model of Morph is quite different from the evolutionary model used here, and it is likely that Morph will interpret any weak match to a PWM, even if not conserved, as a TFBS (often a spurious site), thus inflating the scores of all sequences and making discrimination between the two sets more difficult. Overall, the best performance is obtained by EMMA2 at moderate to high divergence levels. This, combined with the fact that in practice the divergence level for pairwise comparison often lies in this range (e.g. human-mouse divergence is estimated to be 0.6–0.8 [49]), justifies the use of our full model, EMMA2, for practical tasks of CRM prediction. We also repeated the same comparisons using data simulated under CisEvolver, and obtained similar conclusions (data not shown).

In summary, we made several findings through simulation that are relevant to choosing and developing the computational tools for CRM prediction. Generally, one should use relatively divergent sequences, as long as they are alignable. The performance depends on the alignment of TFBSs, but less on the overall alignment quality, or the exact alignment parameters such as gap penalties. When the binding site turnover events are common, it is important to model the lineage specific binding sites. Finally, because of the inherent uncertainty of alignment, simultaneous inference and alignment taking advantage of the special properties of the sequences will help both tasks. All these observations support our efforts in building a comprehensive model of CRM evolution and using it as a basis for related inference tasks.

### EMMA Improves Detection of TFBSs in Fly Developmental CRMs

To test if our model truly brings benefits in real-world applications over existing programs, we start with the task of alignment, and compare our program EMMA (its full version, EMMA2) with Lagan and Morph. We study the set of blastoderm CRMs from the RedFly database [44] in *D. melanogaster* and *D. pseudoobscura*. Since it is not possible to know the true alignments of the real data, we follow the earlier approach [50] of evaluating an alignment by how often a TFBS appears to be conserved in this alignment: a correct alignment should contain more conserved TFBSs on average than an incorrect alignment. We call a TFBS conserved in an alignment if it appears as a gapless block, and both orthologous sites have binding energy above some threshold (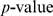 0.002, where 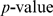 is defined through a standard likelihood ratio score [13],[51]).

**Figure 4:**
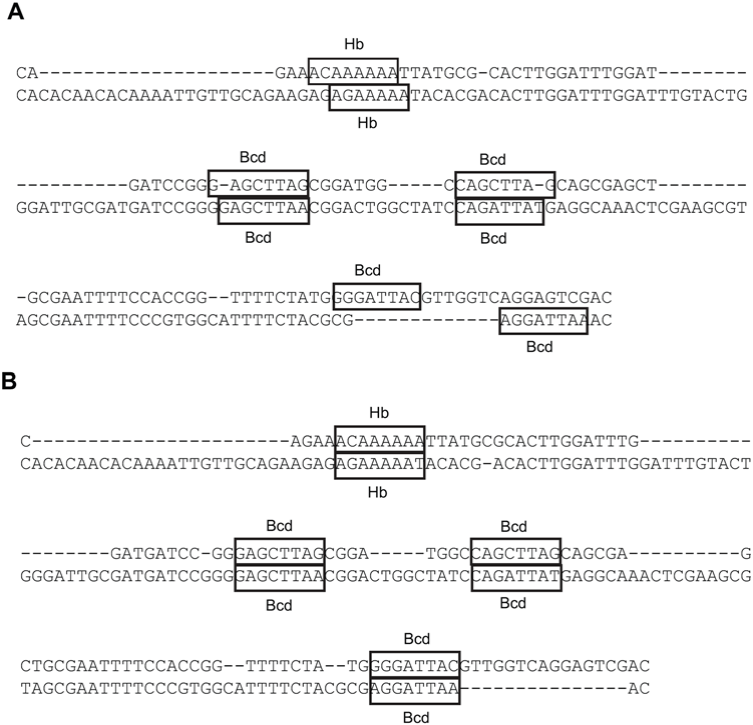
*D. melanogaster*-*D. pseudoobscura* alignment of part of the CRM, “hb anterior activator”. Shown in the *D. melanogaster* sequence (top) are the FlyReg sites of Bcd and Hb, and shown in the *D.pseudoobscura* sequence are the predicted sites in this region. (A) Lagan; (B) EMMA.

In our first experiment, we use the known TFBSs [52] of seven motifs important in the blastoderm stage of development. Among the total of 188 known sites in 65 CRMs, 80 are conserved in the Lagan alignment, while 91 and 103 are conserved in EMMA and Morph alignments, respectively. We further manually examined some sequences on which the alignment programs disagree and show one such example in Figure 4. Three patterns of possible mis-alignment are revealed in Lagan alignment. For the first Hb site, the orthologous site is shifted by two nucleotides likely because the Hb motif has a repeat structure (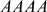 in its consensus sequence). For two Bcd sites in the middle row, the nucleotides at the boundaries are not aligned. In particular for the first one, the gap in *D. melanogaster* can be moved by one position without changing the Lagan score, suggesting that arbitrary resolution of ambiguous alignments can contribute to small-scale alignment errors that may be important for binding sites. Finally, the last Bcd site in *D. melanogaster* is close to, but does not align to a potentially orthologous Bcd site in *D. pseudoobscura*. In EMMA alignment, all four sites in *D. melanogaster* are aligned with their functional orthologs in *D. pseudoobscura*.

We next use predicted sites for further evaluation, since the number of known TFBSs is small. For each of the seven motifs, we constructed alignments with Lagan, EMMA and Morph, using only one motif a time. The results were evaluated by the number of predicted sites (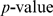 0.002) that appear conserved in the alignments. The results are shown in Table 1. Similar to what we have found above, the number of conserved sites under EMMA is significantly higher than that under Lagan, for all motifs but Kni and Tll. The performance of Morph is intermediate between EMMA and Lagan.

**Table 1:** Number of predicted TFBSs in blastoderm CRMs that are conserved in different alignments.

TF	Bcd	Cad	Gt	Hb	Kni	Kr	Tll
Total	319	412	432	664	293	313	257
Lagan	140	132	102	192	68	100	111
EMMA	166	152	117	244	70	126	115
Morph	154	140	111	220	69	112	112

The “Total” row shows the total number of predicted TFBSs in *D. melanogaster*.

Though our alignment evaluation is not perfect, all the evidence taken together strongly suggests that by utilizing the knowledge of binding motifs, EMMA can significantly improve detection of TFBSs over general purpose tools by overcoming the alignment problems such as arbitrary gap placement. Morph can also improve TFBS detection, but because lineage-specific TFBSs have to be gap-aligned, Morph results do not capture the true evolutionary history of orthologous sequences.

### EMMA Improves Prediction of Regulatory Targets of Fly TFs

In this experiment, we tested different programs for predicting regulatory target sequences of a given TF. Each program was made to score test sequences with a single known PWM. We did not follow the previous procedure [3],[15] of classifying CRM and non-CRM sequences based on sets of known motifs, because we believe that our setting will make the task more challenging and thus make it easier to see the differences of various methods. Furthermore, this experimental setting is particularly relevant to the problem of reconstructing transcriptional regulatory networks, since knowing regulatory relations is often the goal, rather than knowing whether a sequence is a CRM *per se* [53]. In addition to EMMA, Stubb and Morph, we tested Cluster-Buster, a popular CRM finding program [54]. Cluster-Buster uses a HMM to search for binding site clusters in a given sequence and may therefore be used to discover such clusters for individual TFs. Unlike other methods we are testing, Cluster-Buster does not directly use information in orthologous sequences. For each of the seven blastoderm TFs, we constructed a positive set of sequences: those that contain at least one known binding site of this TF in FlyReg; and we used a common set of random noncoding sequences as the negative set. Again, the *D. melanogaster*-*D. pseudoobscura* comparison is used for this experiment. Our evaluation is based on, first, the same AUC measure used for synthetic data; and second, the average sensitivity of programs at high specificity levels. The latter measure is more relevant in practice than AUC because the score threshold is typically chosen to reduce false positive rate to a satisfiable level.

**Figure 5:**
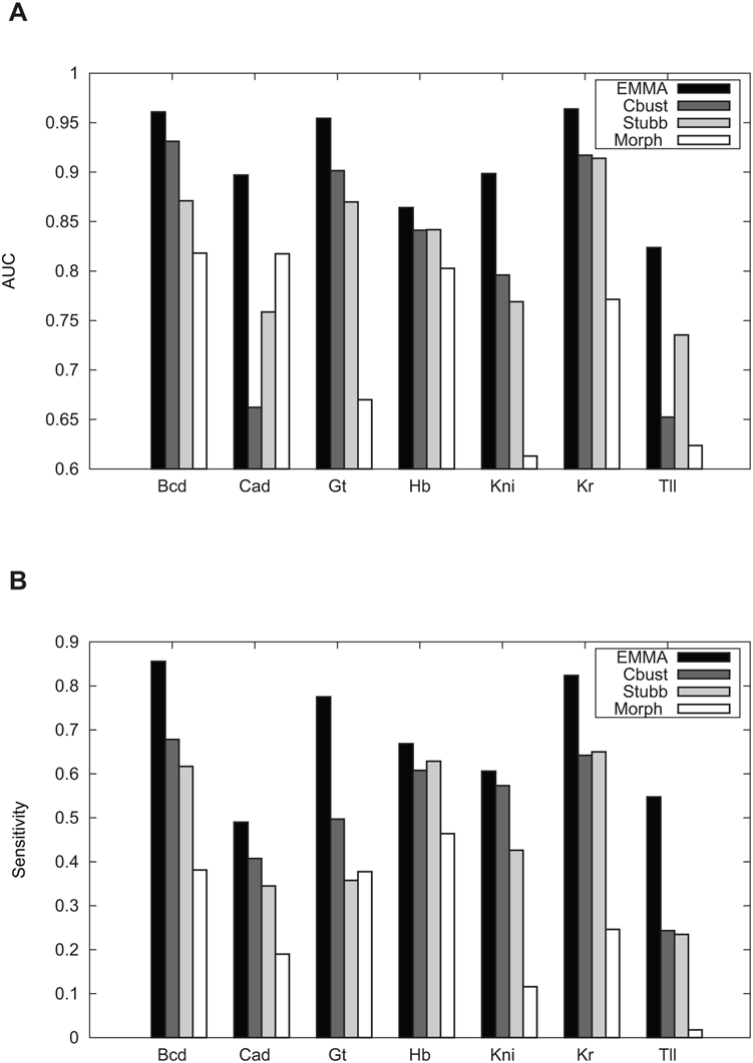
Performances of different programs for predicting regulatory targets of seven blastoderm TFs using *D. melanogaster*-*D. pseudoobscura* comparison. CBust: Cluster-Buster. (A) AUC of the ROC curve; (B) the average sensitivity at the specificity level above 80%.

EMMA substantially outperforms all other three programs with the AUC measure (Figure 5A). Averaging over seven TFs, the improvements of EMMA over Cluster-Buster, Stubb, Morph are 9%, 9% and 17% respectively. Measured by the average sensitivity corresponding to the specificity levels above 80% (Figure 5B), the improvements of EMMA are even more convincing: 15%, 21%, 42%, over the three programs respectively. These results support the key ideas of EMMA: dealing with uncertainty of alignment and explicit modeling TFBS evolution will greatly assist the prediction of regulatory sequences. Interestingly, even though Cluster-Buster uses only sequences in *D. melanogaster*, it is comparable to or even better in some cases than Stubb and Morph, which are based on somewhat similar HMM models and use extra information in the orthologous sequences. Since unlike Cluster-Buster, neither Stubb nor Morph applies a threshold for determining a TFBS, it is likely that they are more sensitive to false positive sites. The problem seems particular serious for Morph because Morph allows a site to be emitted from only one sequence and thus may be overly tolerant to lineage-specific sites matching a PWM (also likely false positive sites). We also note that the experimental setting in this paper is different from the one in [20], where multiple motifs are used simultaneously to classify a sequence. In that setting where there is more motif information and the relative importance of conservation may be reduced, the ability of Morph to score non-conserved weak sites may become an advantage.

One common procedure to enhance the performance of a program running on single-species data, such as Cluster-Buster, is to filter out the sequences that are not very conserved before running the program. We combined this conservation filtering (percent identity greater than 70%, other values of threshold gave similar or worse results) with Cluster-Buster. However, the new results are only slightly better than the original Cluster-Buster, and still lag far behind EMMA (data not shown). This can be probably explained by the fact that a large fraction of *Drosophila* genome is under constraint [55],[56],[57], thus simple conservation measure is not very discriminative of CRM sequences. To test if our results are robust to PWMs, we also repeated the same experiment with PWMs of the same TFs obtained from bacterial one-hybrid experiments [58] and found similar trends (Figure S3).

### Binding Site Conservation in Sequences Bound by Key Transcription Factors in the *Drosophila* Blastoderm

**Figure 6:**
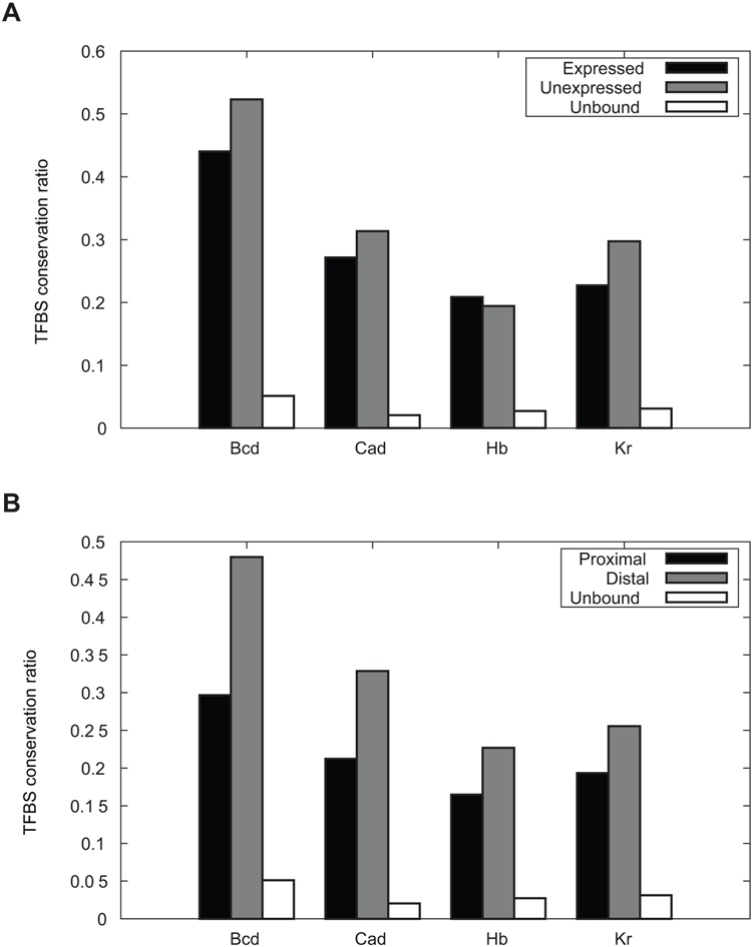
Conservation of predicted binding sites in regions bound by TFs in the *Drosophila* blastoderm. (A) Expressed/Unexpressed: sequences that are associated with genes that are expressed/unexpressed in stage 4–6 according to BDGP; Unbound: randomly chosen intronic sequences. (B) Proximal: sequences that are less than 2 kb from the TSS of the associated genes; Distal: sequences that are more than 10 kb from the TSS of the associated genes.

In this experiment, we used EMMA to study the evolutionary pattern of TFBSs in sequences involved in gene regulation in blastoderm-stage development of *Drosophila melanogaster*. Such analysis depends on the accurate alignment of TFBSs, a task that EMMA has been shown to perform better than general purpose sequence alignment tools. We took the sequences bound by each transcription factor (except Gt and Kni, see Materials and Methods), as per ChIP-chip assays in Li et al. [32]. As a “negative control”, we took the intronic sequences that were not bound by the corresponding TFs. These control sequences are presumably neutral or close to neutral [32]. Each “bound sequence” was associated with its nearest gene. We grouped sequences based on whether their associated genes are expressed in the blastoderm or not. (The expression information was obtained from Berkeley Drosophila Genome Project (BDGP) [59]). The two groups were compared based on the level of conservation of predicted binding sites, defined as the percentage of binding sites in *D. melanogaster* that are conserved in *D. pseudoobscura*. We expect that the sequences in the expressed group are more conserved than those in the non-expressed group, because binding in the latter group is much more likely to be non-functional. Contrary to our expectation, the non-expressed group appears to be have slightly more binding site conservation than the expressed group (Figure 6A), though the difference is not significant (data not shown). Compared with the control sequences, sequences in both groups have much greater binding site conservation, suggesting functional constraint. We next compared the bound sequences that are proximal to TSS (defined as less than 2 kb distant) and those that are distal (defined as greater than 10 kb). Our expectation is that the proximal sequences overall are more functionally important than the distal sequences, as suggested by others [2],[35], and have more conserved binding sites. The results, however, show the opposite pattern (Figure 6B): binding sites in distal sequences tend to be more conserved than those in the proximal ones, and the differences are statistically significant (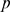 value <10^−4^ for Bcd, Cad, Hb, and <0.005 for Kr, by hypergeometric test).

## Discussion

We have proposed an integrative framework for cross-species analysis of *cis*-regulatory sequences. At the heart of our approach is a probabilistic model covering important aspects of CRM evolution, including substitutions and indels in background sequences, and constraints and turnover of TFBSs. The dynamic programming algorithm allows us to efficiently carry out likelihood-based statistical inference. This framework solves the problems of the existing approaches discussed earlier. It aligns regulatory sequences by taking advantage of the tendency of conservation of TFBSs. The TFBS gain and loss model allows us to use information present in lineage-specific TFBSs. Most importantly, when used for predicting CRMs, our method treats alignment and annotation of TFBSs as random variables, summing over them and thus minimizing the impact of an uncertain alignment and TFBS annotation. Our previous programs Stubb and Morph have similar aims, but as shown in our experiments, EMMA significantly outperforms both, strongly suggesting that correct evolutionary modeling is essential to fully utilize the sequence information.

Our model is related to existing models of regulatory sequence evolution, but different from them in several key aspects. Our idea of generation of a new binding site is similar to [27],[31], but their work is limited to simulation studies. Lassig and colleagues [25],[28] have developed population genetic models where a binding site evolves under a fitness function that depends on the edit distance (to the consensus site) or the energy of the site. Their models are the most detailed and perhaps realistic existing models of binding site evolution; however, they cannot be easily used for computational inference since likelihood computation under these models is very expensive (see below). Mustonen and Lassig [28] also proposed ways to model the gain and loss events of TFBSs, but their model is different from ours in that these events are caused by external selection forces, whose rates of occurrences are independent of the actual sequences. A similar model of TFBS turnover has been used to discover lineage-specific TFBSs [30], where the gain and loss of binding sites are modeled by a two-state Markov chain, similar to the Jukes-Cantor model of nucleotide evolution. Again, the rates of change between functional and neutral sites are external parameters that do not depend on the sequences themselves. Durrett and Schmidt [26] studied binding site evolution from the perspective of time needed for a specific word to appear and be fixed in a population, according to population genetic models of mutation and drift. Their study assumes neutral evolution and points out that selective forces will take over if the specific word thus evolved is close to being a binding site; this is the view we have adopted in modeling binding site gain. Recently, Raijman et al. [29] developed a model of CRM evolution, based on the idea that any mutation that creates a new TFBS or destroys an existing one is penalized, i.e., fixed with a smaller probability. Their representation of TFBSs is based on the consensus sequence, instead of the more realistic PWM. Their treatment of TFBS gain is also different from ours: the possibility of TFBS gain from adaptive selection [13] is missing in their model, where all occurrences of new TFBSs will be selected against. Finally, we note that none of the above models integrates the binding site evolution model with the model of insertions and deletions, a feature that is essential to simultaneous alignment and regulatory sequence inference.

Our model is also an extension of statistical alignment (reviewed in [22]) to the analysis of *cis*-regulatory sequences. Our method shares key features with statistical alignment: explicit modeling of indel evolution; and a probabilistic treatment of alignment uncertainty. Statistical alignment started with the pioneering work of Thorne et al. on pairwise sequence alignment [10], commonly named TKF91 model, where insertions and deletions were treated as single nucleotide events. It was later extended to more realistic indel models, where the indels were treated as multi-nucleotide blocks that followed a geometric length distribution, emulating the commonly used affine gap penalty [23],[37],[60], or an arbitrary length distribution estimated empirically [61]. In other work, the TFK91 model has been applied to multiple alignment, and an MCMC approach developed to sample alignment from a phylogenetic tree [62]. To make the evolutionary model more realistic, some researchers have attempted to capture the heterogeneity of substitution and indel rates and used it to infer slowly-evolving DNA sequences [21],[23],[63]. More recently, the “transducer” model has provided a computational framework for multiple alignment, using TKF91 and other indel models [64],[65]. Our work, especially its alignment functionality, belonging to the category of statistical alignment; however, it is designed specifically for the alignment of *cis*-regulatory modules. Thus the modeling of substitution and indels, the characteristic feature of statistical alignment, has to be integrated with a model-based treatment of binding site evolution.

One main limitation of our model is that under Halpern-Bruno model, the nucleotides of a TFBS evolve independently while in reality, the TFBS as a whole should be a unit for natural selection [28]. Also, our model of TFBS gain and loss does not parameterize the fitness function of a TFBS, which will be required for correct modeling based on principles of population genetics[28],[39]. So our model can be viewed only as an approximation. Our model choice was based on: (i) avoidance of additional free parameters, which will be difficult to estimate given only an individual CRM sequence; (ii) computational complexity, since modeling a TFBS as a unit is very expensive [28],[29]. One consequence of our simplifications is: any new site created by evolution of background sequences will be selected afterward. A better model should reflect the variability of the rate of TFBS gain in different CRM sequences. Despite these simplifications, we found through simulation that the gain and loss rates under our model with a realistic parameter setting agreed broadly with the empirically estimated values in *Drosophila* [13],[66] (data not shown).

The relationship between TF binding and target gene expression is an important, but not straightforward, issue. Earlier studies suggested a high level of non-functional binding in ChIP-chip experiments. Gao et al. estimated that more than 40% TF binding are not functional, based on the correlation of binding and mRNA expression [33]. More recently, Hu et al. found that only a small percentage of genes whose promoters bind to some TF changed expression level when that TF was knocked out in yeast [34]. Our analysis based on binding site conservation provides a new way of studying binding-expression relationship. We find that sequences whose associated genes are not expressed, and thus most likely non-functional, are at least as conserved as the sequences close to expressed genes. This suggests that the extent of non-functional binding may be very low, at least when we restrict ourselves to strong binding events (1% FDR). This immediately raises the following question: if strong binding sites near non-expressed genes are indeed functional (as their evolutionary conservation would reveal), what is this function? We speculate several answers. The function of these sites may be to control expression of more distant genes. (Recall that we annotated only the nearest gene as being the target of each site.) Alternatively, these sites may not directly activate or inactive expression, rather, they help attract TF molecules to DNA, and thus help direct the TF molecules to their true target sequences. Another possibility is that these sites function in regulating the nearby gene in a different developmental stage (i.e., not in blastoderm).

Very little is known about the difference between proximal and distal regulatory sequences. It is likely that the two types of sequences work through different mechanisms (for example, the distal sequences may need specific mechanisms such as DNA looping, to communicate with the core promoter sequences of the target genes [67]) and that they play different functional roles, as hypothesized by [35]. Our results suggest that binding sites are more conserved in the distal regions than in proximal regions. One possible explanation is that the proximal sequences are under more adaptive selection than distal sequences, perhaps because it is easier to achieve a different expression pattern by changing the binding sites in the proximal sequences. This increased adaptive selection has been demonstrated in *Drosophila* in 5′ UTR sequences [55]. Another possibility is that because it is more difficult for distal regulatory sequences to target the promoters, they will be more sensitive to minor changes of binding sites, and thus will be more evolutionarily constrained.

We believe that our proposed framework opens up possibilities for a few major applications. The immediate task is to extend the current work to comparison of more than two species. In pairwise comparison, a TFBS is either conserved or not and it is difficult to distinguish a non-conserved but functional TFBS from a spurious site. In the case of multi-species comparison, there is a wide spectrum of partial conservation, which could be effectively used by a program, as shown in earlier studies [68]. We therefore anticipate that our improved evolutionary model and methods will make a crucial difference to the accuracy of multi-species analysis. Our method takes a set of TF motifs as input; however, which TFs may cooperate while binding is often unknown. Our framework itself offers a way of learning such regulatory rules: the probability of sequences under different TF combinations could suggest how well a particular combination explains the data. Finally, it is possible to learn motifs *de novo* by treating PWMs as unknown parameters. This approach to motif finding will introduce several benefits over existing programs, e.g., PhyloGibbs [6], such as correcting the alignment errors and using information in partially-conserved TFBSs.

## Materials and Methods

### Dynamic Programming Algorithm

We use 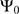 to denote the background evolutionary model (both substitutions and indels), and 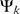 for the evolutionary model for binding sites of the 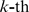 TF (HB model for substitution and reduced indel rate 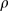). The joint probability of the orthologous sites 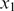 and 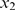 under a model 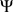 (background or TFBS) is represented as: 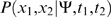, where 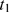, 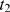 are branch lengths of the 2 sequences. In the case of TFBS gain or loss, the probability of a functional site of 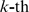 TF being present in the first sequence but not in the second one is denoted by 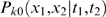; similarly we use 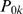 for the opposite case.

**Figure 7:**
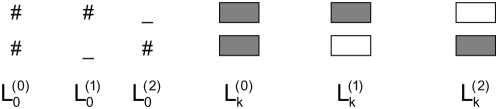
The recurrence variables used in dynamic programming. #: any of the four nucleotides; −: gap; shaded box: TFBS; open box: non-TFBS.

For a pair of sequences 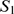 and 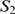, we wish to compute the joint probability of the two sequences under the CRM evolutionary model, given the parameters. We define the recurrence variables for dynamic programming as: 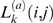, the probability of sub-sequences 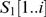 and 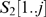 where the last site is either 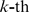 TFBS (if 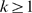) or background (if 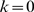) with the “state variable” 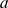 as explained below and in Figure 7. We then define:
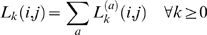
(4)

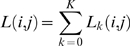
(5)Then the probability of the sequences is 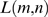 where 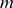 and 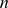 are their respective lengths. In the first case in Figure 7, the last site of the sequence 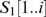 and 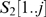 is a matched background column:
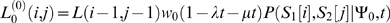
(6)where 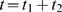. In the second case, the last column is a gap in the second sequence. If the previous column is also a gap, this should be treated as extension of an existing indel, otherwise as a new indel:
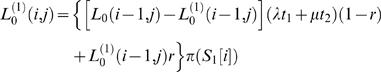
(7)The third case, 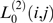, is handled similarly. In the fourth case, the last sites are a conserved pair of TFBSs of the 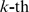 motif, whose length is 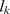:
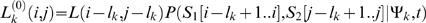
(8)In the fifth case, the last site is a 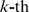 TFBS in 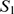 but a non-site in 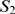. Note that in this case, the length of the non-conserved site may not be 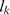, since there could be insertion or deletion in non-TFBS. We will denote it as 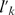. We use 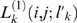 to denote the probability that the site in 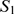 is 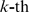 TFBS, but the site in 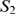 is background with length 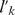, then:
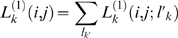
(9)To make the computation tractable, we will limit 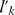 to the range of 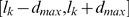 for some user-specified parameter 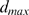 Thus, for the new recurrence variable, we have:
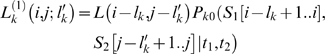
(10)The treatment of the last case is similar. One complication is: if the orthologous sites have a gap at the beginning, it may be an extension of an existing indel. In other words, we may have multiplied the probability of an indel event twice: one during the computation of TFBS switching (the second term in Eq. 10), and the other during the computation of the earlier sequences (the first term in Eq. 10). We correct for this as described in Text S1.

For alignment, we simply need to replace the sum operator in the algorithm with the max operator, as is standard in computations involving HMM. The parameters can be estimated by the standard maximum-likelihood approach. In practice, we estimate or fix some parameters, such as transition-transversion bias in HKY model, through external data. The details can be found in Text S1.

### Simulating CRM Evolution

For our simulation, we first sampled ancestral sequences from the CRM model described in the main text and evolved the sequences in two branches independently for specified lengths of the two branches. Only the two descendant sequences will be used for alignment input. We used PWMs of *Drosophila* TFs from the webpage maintained by Dan Pollard (http://rana.lbl.gov/˜dan/matrices.html), which are based on footprinted binding sites [52]. For the alignment experiment, we used Bcd, Kr and Hb with densities equal to 0.008, 0.009 and 0.005 respectively. The motif thresholds were chosen so that the expected rate of TFBS gain equals to the expected rate of loss through simulating evolution of individual TFBS. For the CRM discrimination experiment using simulated data, we used only Bcd and Hb motifs with lower densities 0.004 for both. We generated 50 pairs of sequences with ancestral sequence length equal to 500 bp at each divergence time for the alignment experiment; and 100 pairs of positive and negative sequences with the same ancestral sequence length at each divergence time for the CRM discrimination experiment. A total of 8 divergence time from 0.1 to 0.8 were sampled. For both experiments, the other parameters took values estimated from earlier studies involving *Drosophila* genomes. The distribution of nucleotides in the background sequences was 0.3, 0.2, 0.2 and 0.3 for A, C, G, T respectively [13]. And from the same study, the transition-transversion bias was 2.0. For the ratio of indels vs substitutions, we used the value (0.225 ) estimated from two close *Drosophila* species, *sechellia* and *simulans* [61], which was evenly split between insertions and deletion in simulation. The length of indels followed geometric distribution with the probability of adding one more nucleotide equal to 0.87 [56]. The rate of indel within TFBS relative to the rate within background sequence was 0.25, from manually inspecting the alignment of eve-stripe 2 CRM in [7].

For the simulation under CisEvolver, we used the same parameters except that the indel lengths are specified by their empirical frequencies [61] instead of approximation by the geometric distribution.

### Alignment Experiment in Fly Developmental CRMs

We took 67 *D. melanogaster* blastoderm CRMs from RedFly database [69], and extracted their orthologous sequences in *D. pseudoobscura* by using the LiftOver tool from UCSC Genome Browser [70]. Two CRMs without *D. pseudoobscura* orthologs were discarded. We used seven TFs important for early development in our analysis: Bcd, Cad, Gt, Hb, Kr, Kni and Tll and the PWMs of these TFs were taken from the same source we used for simulation. Bona fide binding sites were collected from FlyReg [52], after some preprocessing: the sites in FlyReg frequently contain some sequence flanking the true binding sites, so we scanned each FlyReg site with the corresponding PWM and extracted the best match to be used as the “known” binding site for evaluation purposes, rather than the original FlyReg site.

In running EMMA, we set the evolutionary parameters according to the values estimated from earlier studies: divergence between the two species is about 1.5 measured by synonymous substitution (http://rana.lbl.gov/˜dan/trees.html), since the noncoding sequences in general are under a high level of constraint (on average, the intergenic sequences evolve 50–60% more slowly than neutral ones [57]), we rescale the divergence to be 1.5*(1−0.6) = 0.6; the indel parameters, the transition-transversion bias and the equilibrium distribution of nucleotides are all set by the values used in simulation. All other parameters are either default or estimated from data by the program itself. Both Stubb and Morph were run under the default parameters except that the divergence was set at the same value.

### Experiment of Regulatory Target Prediction for *Drosophila* TFs

For each of the seven TFs, we took all RedFly CRM sequences that contain at least one FlyReg site of this TF as the set of positive sequences. Each positive sequence was expanded or truncated so that the length was 1000 bp. 500 random sequences of length 1000 bp each were chosen randomly from the *D. melanogaster* genome as the negative set. The orthologous sequences in *D. pseudoobscura* genome were extracted similarly using the LiftOver tool. EMMA was run under the same parameter setting as in the alignment experiment. Stubb was run under the same divergence value, 0.6 and Morph used the automatically estimated value of divergence (similar performance was obtained if using 0.6 as the divergence). Cluster-Buster was run under the default setting, as we do not have extra data for training parameters of Cluster-Buster.

### Binding Site Conservation Analysis in ChIP-Chip Data

The genome-wide binding data is taken from [32]. We only looked at four factors in this experiment: Bcd, Cad, Hb and Kr. Gt is ignored in this analysis because the PWM of its binding motif is not very specific, and Kni is also ignored because only 35 peaks are identified at 1% FDR level. A bound region is defined as a peak plus 250 bp flanking sequences both upstream and downstream. For the control sequences, we used an equal number of non-first introns, randomly chosen from the *D. melanogaster* genome. The alignment of the sequences with their orthologs in *D. pseudoobscura* were constructed using EMMA. The binding sites in both species were then predicted following the same procedure we used before for the experiment of evaluating alignment performance of EMMA. Similarly, we define a binding site as being conserved if both orthologous sites have scores greater than the threshold (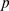 value 0.001). To define expressed and unexpressed group, we used the annotations in BDGP of the expression patterns of genes measured by *in situ* hybridization (http://www.fruitfly.org/cgi-bin/ex/insitu.pl). A sequence belongs to the expressed group, if its associated gene is classfied as being expressed in stage 4–6 according to BDGP, and similarly for the unexpressed group (using the term blastoderm instead of stage 4–6 will give similar results, but we want to be more conservative when defining the unexpressed group).

### Availability

The EMMA program, an evolution simulator, and the dataset used in this paper are all available at http://veda.cs.uiuc.edu/emma/.

## Supporting Information

Figure S1 A pair of orthologous sites that are functional in one species, but not the other. Two possible histories that could lead to this pattern are shown. Shaded and white box represent functional TFBS and non-functional site respectively.
(0.02 MB PDF)

Figure S2 TFBS conservation sensitivity from various alignment programs using simulated data from CisEvolver.
(0.02 MB PDF)

Figure S3 Performances of different programs for predicting regulatory targets of seven blastoderm TFs using B1H PWMs. CBust: Cluster-Buster. (A) AUC of the ROC curve; (B) the average sensitivity at the specificity level above 80%.
(0.02 MB PDF)

Text S1 Methods and additional results
(0.07 MB PDF)
