# Supplementary figures and images for: Alignment and Prediction of cis-Regulatory Modules Based on a Probabilistic Model of Evolution
Source: PLoS Comput Biol. 2009 Mar 13;5(3):e1000299. doi: 10.1371/journal.pcbi.1000299 (PMC2657044; doi:10.1371/journal.pcbi.1000299)

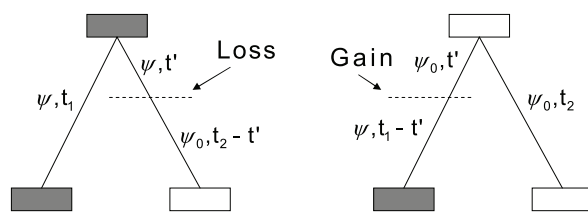

Supplement: Figure S1 — A pair of orthologous sites that are functional in one species, but not the other. Two possible histories that could lead to this pattern are shown. Shaded and white box represent functional TFBS and non-functional site respectively. (0.02 MB PDF) [file pcbi.1000299.s001.pdf]

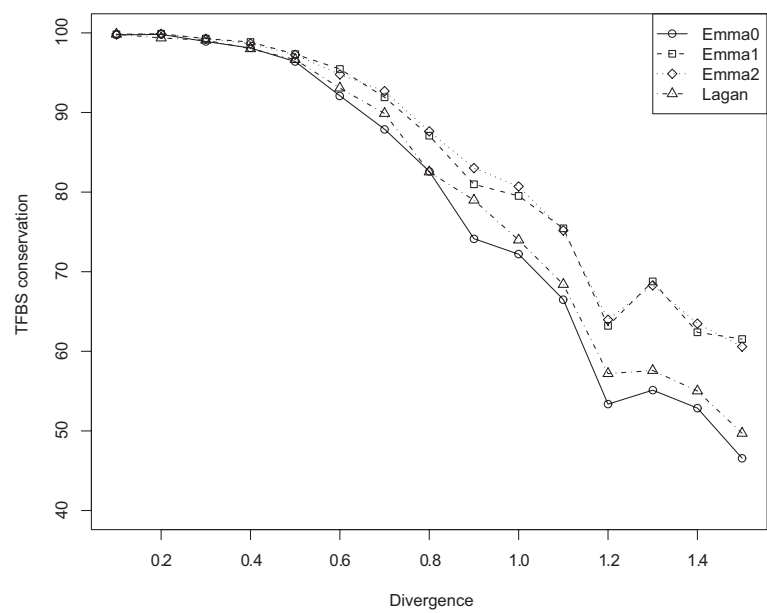

Supplement: Figure S2 — TFBS conservation sensitivity from various alignment programs using simulated data from CisEvolver. (0.02 MB PDF) [file pcbi.1000299.s002.pdf]

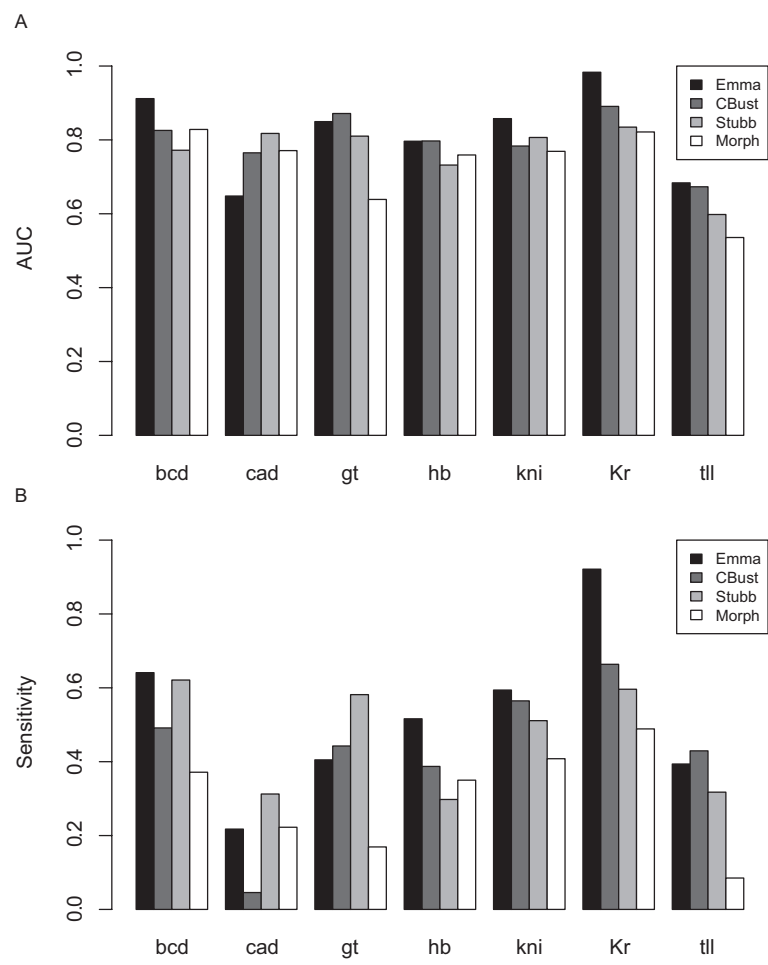

Supplement: Figure S3 — Performances of different programs for predicting regulatory targets of seven blastoderm TFs using B1H PWMs. CBust: Cluster-Buster. (A) AUC of the ROC curve; (B) the average sensitivity at the specificity level above 80%. (0.02 MB PDF) [file pcbi.1000299.s003.pdf]
