# Supplementary material for: Alignment and Prediction of cis-Regulatory Modules Based on a Probabilistic Model of Evolution
Source: PLoS Comput Biol. 2009 Mar 13;5(3):e1000299. doi: 10.1371/journal.pcbi.1000299 (PMC2657044; doi:10.1371/journal.pcbi.1000299)
Supplement: Text S1 — Methods and additional results (0.07 MB PDF) [file pcbi.1000299.s004.pdf]

Supporting Information for: Alignment and Prediction of cis-Regulatory Modules Based on a Probabilistic Model of Evolution

Xin He, Xu Ling, Saurabh Sinha

1 Computing indel probabilities

To compute the transition probability of indels, we make two simplifications that have often been made, for example [1]: first, the insertion and deletion rates are low so that the probability of an insertion event in time t is roughly λt (instead of the exact value $1-e^{-\lambda t}$), and similarly for deletion events; second, we ignore possible "multiple-hits" at one position, in other words, we will explain any observed indel as created from a single insertion or deletion event. Consider the example in the main text:

```
x:  ####---#
y:  #--#####
```

Under a two-species phylogenetic tree with branch lengths t_1 and t_2, the probability of x becoming y in time t is:

$$P(x \rightarrow y|t) = (1 - \lambda t - \mu t)^3 \lambda t(1 - r)r^2 \mu t(1 - r)r \tag{1}$$

The term $(1-\lambda t-\mu t)$ is the probability of not seeing an insertion or deletion event in t. To compute the joint probability of both sequences in the case of pairwise comparison, the ancestral sequence must be summed out since it is not observed. Specifically, consider a simple two-species phylogenetic tree with branch lengths t_1 and t_2 respectively, we wish to compute the joint probability of a single indel ($\#\#, --$). There are two cases: the ancestor is a gap then the indel is due to insertion in the first branch; or the ancestor is nucleotides, then the indel is due to deletion in the second branch. Suppose the probability of planting an extra nucleotide in the ancestral sequence is p, then:

$$P(\#\#, -- |t_1, t_2) = \lambda t_1(1 - r)r + p^2 \mu t_2(1 - \lambda t_1 - \mu t_1)(1 - r)r \approx (\lambda t_1 + \mu t_2)(1 - r)r \tag{2}$$

where we made the assumption that both p and $(1 - \lambda t_1 - \mu t_1)$ are close to 1 (the ancestral sequences are generally long, so p should be close to 1, and indel events are relatively rare). The joint probability of the example given above involving multiple indels is thus approximately:

$$P(x, y|t_1, t_2) = (1 - \lambda t - \mu t)^3(\lambda t_1 + \mu t_2)(1 - r)r^2(\mu t_1 + \lambda t_2)(1 - r)r \tag{3}$$

where $t = t_1 + t_2$.

2 Computing probabilities of lineage-specific TFBS in a two-species tree

In a two-species phylogenetic tree, the observation of a functional site in the first sequence, but a non-functional orthologous site in the second can be interpreted as: a loss event in the second

branch or a gain event in the first branch. As illustrated in Figure S1, the joint probability is given by:

$$\begin{aligned} P(x_1, x_2|t_1, t_2) = &\sum_{(z,z')} \int_0^{t_2} P(x_1, z|\Psi, t_1, t')Q(z, z')P(z' \to x_2|\Psi_0, t_2 - t')dt' \\ &+ \sum_{(z,z')} \int_0^{t_1} P(z' \to x_1|\Psi, t_1 - t')Q_0(z, z')P(z, x_2|\Psi_0, t', t_2)dt' \end{aligned} \tag{4}$$

The meaning of the variables, the energy and neighborhood constraints (z, z') should satisfy, have all been defined in the main text.

3 Correction of dynamic programming

As described in the main text, when an indel crosses the boundary between a TFBS region (one of the two orthologous sites is a TFBS, but the other not) and the neighboring background sequence, we need to correct our recurrence equation. The idea is that we need to divide the probability of opening an indel so that it is multiplied only once in the computation. The new equation for the recurrence variable $L_k^{(1)}(i, j; l'_k)$ will be:

$$\begin{aligned} L_k^{(1)}(i, j; l'_k) = &\{[L(i - l_k, j - l'_k) - L_0^{(1)}(i - l_k, j - l'_k)] \\ &+ L_0^{(1)}(i - l_k, j - l'_k)\tfrac{1}{(\lambda t_1 + \mu t_2)(1-r)}\} \\ &\cdot P_{10}(S_1[i - l_k + 1..i], S_2[j - l'_k + 1..j]|\Psi_k, \Psi_0, t_1, t_2) \end{aligned} \tag{5}$$

4 Parameter estimation

All the parameters used by the program are listed in Table S1. In theory, we can estimate all parameters, except the switching threshold for each TF, by the standard maximum-likelihood approach. In practice, we offer the options of using estimated values from external data or from heuristic approaches. Specifically, for the background nucleotide distribution π, we will set it as the frequencies in the input sequences. For the substitution parameters of the background sequences (we assume that the divergence time is 1 and only need to estimate the background rate since the two are not separable in HKY model), we offer two options: either align the sequences with a general tool like LAGAN and estimate the rate and bias from the program PAML [2]; or use the estimated values from previous genome-wide studies. For the background indel parameters λ, μ and r, we again obtain them from previous studies, or estimate them in the following way: we first align the sequences with LAGAN, then set the rates λ and μ so that the expected fractions of indels are equal to the observed amounts, that is:

$$\begin{cases} \lambda t_1 + \mu t_2 = f_1 \\ \mu t_1 + \lambda t_2 = f_2 \end{cases} \tag{6}$$

where f_1 and f_2 are the fractions of the two types of gaps: $(\#, -)$ and $(-, \#)$ respectively. The parameter r is similarly set so that the expected and observed average indel length are equal:

$$\overline{n} = \frac{1}{1 - r} \tag{7}$$

The intra-TFBS indel rate ρ is trained from external data since in general, it cannot be reliably estimated from an input pair of sequences. We set the default value be 0.25, by manually inspecting the alignment of eve-stripe 2 CRM in [3]. The weight parameters $w_k, 1 \leq k \leq K$ are estimated from maximum-likelihood approach. The switching threshold for each TF is determined by using a p value cutoff: at default value $p = 0.002$, the threshold is chosen at the binding energy of

the top 0.2% among all sites generated by sampling from a specified nucleotide distribution. Our default distribution for energy computation is $(0.3, 0.2, 0.2, 0.3)$ for (A,C,G,T), which is the global nucleotide frequencies in *D. melanogaster* [4].

References

1. Siepel A and Pollard KS and Haussler D (2006) New methods for detecting lineage-specific selection. Proceedings of the 10th International Conference on Research in Computational Molecular Biology:190-205.

2. Yang Z (2007) PAML 4: Phylogenetic Analysis by Maximum Likelihood. Mol Biol Evol, 24(8):1586-91.

3. Ludwig M, Patel N, Kreitman M (1998) Functional analysis of eve stripe 2 enhancer evolution in *Drosophila*: rules governing conservation and change. Development 125:949–958.

4. Moses A, Pollard D, Nix D, Iyer V, Li X, et al. (2006) Large-scale turnover of functional transcription factor binding sites in *Drosophila*. PLoS Comput Biol 2:e130.

5. Noyes M, Meng X, Wakabayashi A, Sinha S, Brodsky M, et al. (2008) A systematic characterization of factors that regulate Drosophila segmentation via a bacterial one-hybrid system. Nucleic Acids Res 36:2547–2560.

Table S1: Parameters used by the program Emma.

w_k	weight of the k-th motif in CRM ($k = 0$: background)
l_k	length of the k-th motif ($1 \leq k \leq K$)
θ_k	the PWM of the k-th motif ($1 \leq k \leq K$)
π	stationary distribution of nucleotides in the background
α	the substitution rate of the background
β	transition-transversion bias of the background
λ	insertion rate in the background
μ	deletion rate in the background
r	length distribution of indels in the background (probability of extension)
ρ	rate of indels within TFBS relative to the rate in the background

Table S2: Specificity of alignment programs on simulated data. The values after / sign are the performance relative to Emma0.

Div.	Emma0	Emma1	Emma2	Lagan	Morph
0.1	98.21/0	98.25/0.04	98.20/-0.01	97.94/-0.27	99.00/0.79
0.2	92.73/0	92.87/0.14	92.81/0.08	91.04/-1.69	94.95/2.22
0.3	84.81/0	85.10/0.29	85.02/0.21	82.65/-2.16	88.17/3.36
0.4	73.23/0	73.77/0.54	73.60/0.37	71.46/-1.77	75.34/2.11
0.5	58.92/0	60.55/1.63	59.71/0.79	57.14/-1.78	59.47/0.55
0.6	43.17/0	45.07/1.9	44.33/1.16	42.89/-0.28	39.71/-3.46
0.7	30.75/0	34.15/3.4	32.09/1.34	30.58/-0.17	27.90/-2.85
0.8	24.55/0	27.93/3.38	26.49/1.94	25.73/1.18	21.96/-2.59

Table S3: Sensitivity of alignment programs on simulated data. The values after / sign are the performance relative to Emma0.

Div.	Emma0	Emma1	Emma2	Lagan	Morph
0.1	98.52/0	98.55/0.03	98.50/-0.02	98.52/0	98.16/-0.36
0.2	94.33/0	94.43/0.1	94.35/0.02	93.93/-0.4	93.26/-1.07
0.3	87.93/0	87.90/-0.03	87.84/-0.09	87.29/-0.64	85.61/-2.32
0.4	78.26/0	78.57/0.31	78.38/0.12	78.41/0.15	75.20/-3.06
0.5	66.40/0	67.75/1.35	67.20/0.8	66.13/-0.27	61.70/-4.7
0.6	50.89/0	52.34/1.45	52.31/1.42	52.21/1.32	44.06/-6.83
0.7	38.37/0	42.15/3.78	39.70/1.33	38.80/0.43	32.20/-6.17
0.8	32.81/0	36.58/3.77	34.91/2.1	34.49/1.68	25.98/-6.83

Table S4: Specificity of alignment programs on simulated data from CisEvolver. The values after / sign are the performance relative to Emma0.

Div.	Emma0	Emma1	Emma2	Lagan
0.1	98.82/0	98.82/0	98.81/-0.01	98.36/-0.46
0.2	95.60/0	95.59/-0.01	95.55/-0.05	95.01/-0.59
0.3	88.59/0	88.78/0.19	88.74/0.15	87.83/-0.76
0.4	80.79/0	81.19/0.4	81.14/0.35	79.95/-0.84
0.5	69.91/0	70.69/0.78	70.97/1.06	69.97/0.06
0.6	56.58/0	58.37/1.79	58.27/1.69	57.84/1.26
0.7	47.68/0	49.55/1.87	49.34/1.66	48.27/0.59
0.8	40.08/0	42.06/1.98	42.15/2.07	40.40/0.32
0.9	32.26/0	35.41/3.15	36.36/4.1	35.62/3.36
1.0	26.38/0	29.50/3.12	29.66/3.28	27.37/0.99
1.1	22.18/0	25.52/3.34	25.77/3.59	23.38/1.2
1.2	16.14/0	20.04/3.9	20.82/4.68	18.55/2.41
1.3	15.73/0	21.40/5.67	21.07/5.34	17.13/1.4
1.4	13.59/0	17.18/3.59	17.24/3.65	14.32/0.73
1.5	10.46/0	15.49/5.03	15.46/5	11.09/0.63

Table S5: Sensitivity of alignment programs on simulated data from CisEvolver. The values after / sign are the performance relative to Emma0.

Div.	Emma0	Emma1	Emma2	Lagan
0.1	98.89/0	98.90/0.01	98.89/0	98.52/-0.37
0.2	95.98/0	95.97/-0.01	95.93/-0.05	95.67/-0.31
0.3	89.78/0	89.85/0.07	89.89/0.11	89.32/-0.46
0.4	82.73/0	83.14/0.41	83.08/0.35	82.11/-0.62
0.5	71.99/0	72.58/0.59	72.91/0.92	72.41/0.42
0.6	59.67/0	61.44/1.77	61.28/1.61	61.07/1.4
0.7	51.09/0	52.78/1.69	52.58/1.49	51.83/0.74
0.8	43.31/0	45.07/1.76	45.01/1.7	43.73/0.42
0.9	35.97/0	39.27/3.3	40.31/4.34	39.54/3.57
1.0	29.84/0	33.12/3.28	33.22/3.38	30.87/1.03
1.1	25.09/0	28.72/3.63	28.86/3.77	26.82/1.73
1.2	18.78/0	23.27/4.49	24.15/5.37	21.73/2.95
1.3	18.36/0	24.79/6.43	24.25/5.89	20.01/1.65
1.4	16.75/0	20.82/4.07	20.95/4.2	17.66/0.91
1.5	13.03/0	18.80/5.77	18.65/5.62	13.95/0.92
